# High Pressure Processing of Bivalve Shellfish and HPP’s Use as a Virus Intervention ^†^

**DOI:** 10.3390/foods3020336

**Published:** 2014-06-11

**Authors:** David H. Kingsley

**Affiliations:** Food Safety and Intervention Technologies Research Unit, Agricultural Research Service, U.S. Department of Agriculture, James W.W. Baker Center, Delaware State University, Dover, DE 19901, USA; E-Mail: David.Kingsley@ars.usda.gov; Tel.: +1-302-857-6406; Fax: +1-302-857-6451

**Keywords:** seafood safety, high pressure processing, bivalve shellfish

## Abstract

Bivalve shellfish readily bioconcentrate pathogenic microbes and substance, such as algal and dinoflagulate toxins, fecal viruses and bacteria, and naturally present vibrio bacteria. High pressure processing (HPP) is currently used as an intervention for *Vibrio vulnificus* bacteria within molluscan shellfish and its potential to inactivate food-borne viruses and bacteria are discussed. Mechanisms of action of high pressure against bacteria and viruses, as well as how time of pressure application, pressure levels, and pre-pressurization temperature influence inactivation are described. Matrix influences such as ionic strength are noted as important additional considerations. The potential of HPP to influence spoilage and enhance shelf-life of shucked shellfish is also discussed.

## 1. Introduction

High pressure processing (HPP) is an increasingly popular method of separating shellfish meat from the shell for molluscan shellfish (*i.e.*, oysters and clams) as well as crustacea (*i.e.*, crabs and lobsters). HPP shucking of shellfish results in completely intact meat and can be a considerable labor saving method [1]. Also HPP-treated shellfish can be opened easily by restaurant staff and does not require a dedicated onsite shucker (*i.e.*, raw bar). Furthermore a consumer survey has demonstrated that while consumers are not particularly knowledgeable about the concept of high pressure processing, they are apparently not resistant to eating foods treated by high pressure [2]. 

The pressures ordinarily used for food processing range from 200 to 600 megaPascals (MPa). One MPa is approximately equal to 9.87 atms of pressure and approximately 145 pounds per square inch (PSI). By comparison, the pressure at the bottom of the Marianas Trench in the Pacific is approximately 100 MPa. Essentially, HPP is a non-thermal process in that foods are not specifically heated. Bivalve shellfish have a number of potential food safety issues associated with their ability to bioconcentrate toxic chemicals and pathogens from water. These include human intestinal viruses and some fecal bacteria from wastewater and sewage, bacteria that are naturally present in estuarine waters, and toxins derived from plankton and dinoflagulates, which can have serious neurologic consequences for shellfish consumers. 

### 1.1. Toxins

Harmful algae blooms and ocean dinoflagulates can produce a number of chemical toxins that can have rather nasty neurological properties. Examples include saxotin, paralytic shellfish poisoning, and amnesiac shellfish poisoning [3,4,5,6,7,8,9,10,11], the net result of which can be brain or motor nerve damage or even death. Testing for these agents is performed using GC-mass spectrophotometry [12] or bioassay using mice [10]. These toxins are thermally resistant, so even thorough cooking will not inactivate them. Unfortunately HPP is not believed to have any discernable effect on these toxins since HPP does not alter chemical bonds [13].

### 1.2. Vibrio

These bacteria are naturally found in abundance within warm tropical waters year round and in temperate waters during the summer season [14,15]. The most serious of these is *Vibrio vulnificus* (*Vv*) which is a substantial problem for the oyster industry. For ordinary persons with no underlying health issues, this bacterium does not pose a serious threat. However for the approximately 7% of the US population that have liver problems due to excessive alcohol consumption, hepatitis, or have diabetes, are immunocompromised, or have elevated blood iron levels, contracting a *Vv* infection carries a 50% risk of mortality [16,17,18]. While perhaps less than one hundred people contract this annually in the US by various routes of infection, there are as many as 10–20 mortalities associated with consumption of shellfish contaminated with *Vv* [19]. A second bacterium, *Vibrio parahaemolyticus* (*Vp*) is associated with oyster consumption, typically causes mild to moderate gastrointestinal illness which ordinarily persists for a few days but can last for several weeks [20]. While quite unpleasant, *Vp* is not associated with a high mortality rate.

Risk of contracting *Vv* and *Vp* by consumption of raw shellfish becomes substantially greater as increasing amounts of these of bacteria are consumed. Vibrio levels are known to increase dramatically after harvest in response to the shellfish being out of the water and in a warm environment [21,22,23,24,25]. As a result, many regulatory authorities require that loads of harvested shellfish be shaded from direct sunlight, harvested at cooler times of the day or even after dark, and mandate cooling protocols that cool shellfish to less than 10 °C within a few hours of harvest to reduce outgrowth of Vibrio bacteria [26,27]. These efforts have reduced the incidence of oyster-associated illness but these measures have not completely eliminated them. As an additional precaution, post-harvest processing is now required for *Vv* in some jurisdictions. [28]. Post-harvest *Vv* interventions used currently include irradiation [29], quick-freeze [30,31], flash heat [32] and HPP [33,34]. 

## 2. Fecal Bacteria and Sanitary Standards for Shellfish

Oysters were first recognized as vectors for typhoid fever in New York around the turn of the 20th century when market shellfish were being exposed to sewage effluent from the city [35]. Today, typhoid fever associated with raw shellfish consumption is virtually unheard of due to sewage treatment, institution of fecal coliform (FC) standards, and its limited circulation among the general public. Currently two separate, but conceptually similar FC standards are used in the US and EU [36,37]. The US classifies its shellfish growing waters based on regular testing to determine the levels of FC bacteria in waters, essentially measuring hygienic water quality. Other localities, such as the EU, measure the amount of fecal coliforms found directly within shellfish from different harvest locations, essentially measuring the quantity of FC bacteria found directly within oysters. With some caveats, these standards do a reasonable job of measuring human and animal fecal impact on growing areas and identifying areas where shellfish can be and should not be harvested [38]. That said, the potential to contract *Salmonella enterica* infections from raw oysters remains a qualified concern. In the US, one report indicated salmonella bacteria in US market shellfish had an incidence of 7.8% [39], but a subsequent report put this number at about 1% [40]. In addition, a recent report seems to indicate that salmonella is able to persist within live shellfish more efficiently than other coliform bacteria [41].

## 3. HPP as a Microbiologic Intervention

### 3.1. Bacteria and Spoilage

Generally Gram-negative spoilage bacteria are more sensitive to HPP than Gram-positive bacteria. For example, the Gram-positive bacterial population fraction within oysters were reported to increase from 56% to 84% Gram-positive after a 500 MPa treatment and storage at 2 °C for 28 days [42,43]. Because much of the typical off-odor associated with spoilage is due to the growth of Gram-negative bacteria, HPP is capable of extending the refrigerated shelf-life of oysters [44]. After HPP, a prominent reduction of bacterial diversity also occurs. For example after a 500 MPA treatment of oysters (*Crassostrea gigas*) and 28 days of storage at 7 °C Linton *et al.* [42] reported that 96% of bacterial isolates were limited to *Bacillus*, *Acintobacter*/*Moraxella* and lactic acid bacteria.

### 3.2. Spores and Fungi

He *et al.* [44] report that many fungi are inactivated by pressures ranging from 300 to 600 MPa. Bacterial spores are a challenge for HPP, although spore reduction has been noted by double-cycle HPP treatment in which pressure is applied, the pressure is released for a brief period to permit spore germination, and then reapplied to kill germinated spores [45]. Unfortunately some viable spores are able to survive these double-cycle treatments.

### 3.3. Vibrio

HPP is highly effective in reducing *Vv* in oyster meat to “non-detectable levels” when approximately 275–300 MPa are applied for 3 min at ambient temperatures [33,34]. While processing interventions are not currently mandated for *Vp* in the US, indications are that pressures slightly above 300 MPa can be effective as an intervention for raw shellfish [33,46,47]. 

### 3.4. Fecal Bacteria

Current commercial HPP treatments for oysters are not thought to be high enough to substantially reduce salmonella, but treatments >350 MPa in culture media did generally show 3-log_10_ reductions of *Salmonella enterica* [48], suggesting HPP has potential for inactivating this bacteria in shellfish. Other bacteria such as campylobacter [49], and shigella [50] are occasionally implicated in oyster-vectored food outbreaks. HPP is capable of inactivating these bacteria, but like salmonella, exact conditions required for inactivation within live shellfish have yet to be defined. Many of the food-borne bacteria found in shellfish are Gram-negative, which as stated earlier, are more susceptible to pressure inactivation than Gram-positive bacteria [51] 

### 3.5. Viruses

Viruses remain a vexing problem for the shellfish industry. There are a large number of pathogenic viruses that can be shed from the human gastro-intestinal tract [52]. These viruses can enter shellfish growing areas as a result of sewage overflows and floods, defective septic systems, overboard waste discharge, or even as a result of a vomiting event [53]. Enteric viruses are shed at high levels, perhaps billions of particles per illness, and are typically highly infectious, with only a handful of particles required to establish an infection [54]. Viruses are very stable in the environment, and can persist in shellfish growing waters and even within shellfish for extended periods [55,56]. Because these viruses require a human gut to replicate, they do not grow, or amplify, within shellfish as a result of temperature abuse as can occur for bacteria. Rather they simply contaminate shellfish as a result of filter feeding activities, typically sequestering themselves at relatively low levels. Because direct testing for viruses is currently impractical, classification of shellfish harvest areas are regulated based on the levels of fecal bacteria in growing waters or directly within the shellfish meats, as described previously. While this classification system prevents a great deal of unsanitary shellfish from potentially reaching the dinner table, it is now recognized that low FC levels in growing waters, or within shellfish meats, do not necessarily indicate that there are no viruses present [57]. This is principally due to the fact that viruses can persist within shellfish tissues for longer periods than FCs [55,56,58]. Presumably this is due to virus’ resistance of the acidic digestive processes of shellfish [56]. Thus, a suitable intervention for viruses potentially sequestered within raw shellfish would be of key significance.

Cooking and depuration, two traditional means of sanitizing shellfish, are of limited effectiveness against viruses. Depuration is a method in which live shellfish are placed in clean water for 2–3 days to permit the bivalves to pump and purge pathogens. This method is generally effective against fecal bacteria which can be reduced by several orders of magnitude, but it is accepted that pathogenic human viruses do not purge efficiently enough to make the process a viable intervention for virus contamination. Cooking is thought to inactivate viruses to a substantial degree, but it is unclear what temperatures and cooking times are completely effective against these viruses within shellfish. In fact, several documented outbreaks have been associated with “properly cooked shellfish” [59,60,61]. Furthermore, consumers often prefer uncooked or lightly-cooked shellfish.

Although there are many different types of fecal viruses that can be potentially transmitted by shellfish, the two principal shellfish-borne virus threats are recognized to be human norovirus (HuNoV) and hepatitis A virus (HAV). Human norovirus is now arguably considered to cause the majority of food-borne incidents worldwide [19,62]. Approximately half of the food-borne incidents associated with shellfish are due to norovirus and the overall fraction of food-borne noroviruses attributed to molluscan shellfish is approximately 13% [63,64].

Initial research on the potential of HPP to inactivate norovirus focused on genetically-related surrogate research viruses, such as feline calicivirus [65,66,67,68] and murine norovirus [69], because human norovirus strains have not been reproducibly propagated in the laboratory [70,71] and there are no suitable small animal research models for the virus. Surrogate work pointed to reasonable prospects for inactivation of norovirus. Feline calicivirus was found to be highly sensitive to HPP with 5 min room temperature treatments of 275 MPa being sufficient to inactivate 7-log_10_ of the virus [68,72]. The subsequent isolation and discovery of murine norovirus, which was propagable, made it possible to evaluate a closer genetic relative of human norovirus [73]. Results indicated that higher pressures, on the order of 400 MPa, were needed to inactivate substantial quantities of this virus [69]. However successful inactivation of murine norovirus within oysters was demonstrated at this pressure level [69,74]. Also work with immunocompromised mice confirmed that inactivation by HPP *in vitro*, as assessed by tissue culture and *in vivo*, as assessed using mice were essentially equivalent [75]. 

More recent work has looked at the potential of HPP to inactivate human norovirus. A human volunteer study evaluated conditions required to inactivate the prototype norovirus strain (GI.1 Norwalk). Four-log_10_ PFU of Norwalk virus was injected into oysters and three 5-min pressure treatments were performed at 400 MPa, 22 °C; 400 MPa, 6 °C; and 600 MPa, 6 °C. Unfortunately only the 600 MPa treatment was sufficient to protect all volunteers [76]. Based on a reduced illness frequency, it was postulated that the 400 MPa, 6 °C treatment may have inactivated some norovirus virus. Subsequent investigations using the newly developed porcine gastric mucin binding assay (PGM-MB) binding assay, which can assess norovirus inactivation, have confirmed that norovirus is sensitive to HPP at about 400 MPa [77,78,79].

HAV contamination of shellfish is now uncommon in most parts of the developed world due to improved hygienic standards and vaccination campaigns [80] but it remains a problem in the developing world and the Mediterranean region [81]. HAV illness can be quite serious, often resulting in hospitalization and occasionally in mortality. Morbidity and mortality due to HAV is often age related, with persons over the age of 50 being more prone to mortality and with young children often having only unapparent infections [82,83]. Considering that exposure induces immunity that is generally thought lifelong, HAV vaccination campaigns are often not given much emphasis in endemic regions since the local populations largely become immune at young ages. Unfortunately shellfish and uncooked fruits and vegetable products grown in endemic regions and sold in developed countries can become vectors for HAV outbreaks [84,85]. A tissue culture-adapted strain of HAV has been evaluated for sensitivity to HPP. Results indicate that a 5 min room temperature treatment at 450 MP is sufficient to inactivate 7-log_10_ of HAV virus stock [68]. Evaluation of HAV-contaminated oysters demonstrated a 3-log_10_ reduction after a 1 min-400 MPa treatment at 9 °C [86]. 

It was hoped that HPP would also inactivate other pernicious viruses which could potentially contaminate shellfish such as Aichi virus [87], hepatitis E virus (HEV; [88]), coxsackie viruses, *etc*. HEV has yet to be evaluated but Aichi and a number of other members of the picornavirus family have proven more tolerant to high pressure, requiring either pressures well above 400 MPa, or even being completely as resistant to 600 MPa treatments [89,90]. 

### 3.6. Parameters

Research has shown that there are a number of considerations for inactivating bacteria and viruses with HPP. Of course the primary determinant for pathogen inactivation is the pressure level applied, which generally follows first-order kinetics since plotting log_10_ pathogen reduction *versus* increasing pressure applied gives a straight line. Beyond pressure levels applied, time under pressure and pre-pressurization temperature can have a considerable influence on inactivation levels. For all pressure-sensitive viruses tested to date, increased application time does increase the amount of virus inactivation observed but the amount of increase observed asymptotically decreases, matching log-logistic or weibull kinetics [43,52,91]. Solutes, such as salt and sugar generally decrease the effectiveness of HPP inactivation for viruses and bacteria [43,51,67,68,92]. Formally speaking the reason for this is unknown, but presumably, the presence of solutes may tend to prevent compression and addition of more water molecules into the solvation cage surrounding the protein. Although currently undefined, this may be an important consideration for shellfish grown in different salinities, since the salt content of bivalves mimics the waters from which they have been harvested. Also, generally speaking, bacteria that are actively growing in exponential phase are more sensitive to pressure than bacteria in stationary phase [51].

Perhaps more intriguing is the concept that temperature has a substantial influence on inactivation. For vegetative bacteria, pressure applied above and below room temperature generally appears to enhance inactivation [51]. For viruses, the temperature effect is variable since different viruses react differently for HPP at different temperatures. For noroviruses and all caliciviruses tested to date, refrigeration temperatures dramatically enhance inactivation, often by several logs [52,69]. Curiously, HAV is the reverse. Room temperature and above dramatically enhance inactivation by HPP as compared to refrigeration temperatures [92,93]. Unlike the *Caliciviridae*, other picornaviruses have shown variable inactivation with respect to temperature [90]. Why viruses behave differently under pressure at different temperatures is currently unknown.

Presumably, HPP-treated shellfish would have a neutral pH, but for other food matrices, pH is another important consideration. Low pH generally enhances HPP inactivation of vegetative bacteria, but viruses respond differently to low pH under pressure. For human norovirus, low pH appears to be inhibitory for HPP inactivation [78]. For HAV, a virus that is known to be tolerant of pH 1, lower pH actually enhances HPP inactivation [92,94]. It is also important to note that weak organic acids, such as acetic acid, become stronger acids under pressure. Why HAV and HuNoV behave differently under pressure in acidic pH is currently unknown.

There can be a substantial temperature increase associated with the application of pressure. As anyone who has ever filled a scuba tank knows, when air inside the tank is compressed, heat is generated, making the tank warm. Likewise, anyone who has fully opened the valve of a pressured tank knows that as the pressure is released, the tank becomes quite cold. These effects are due to a principle called adiabatic heating and cooling [95]. Although the incremental amount of heating/cooling does vary somewhat with the initial temperature at which pressure is applied, the temperature increase is approximately 3–3.5 °C per 100 MPa for water-based commercial units. Also, the degree to which this heat dissipates to the environment varies with the size of HPP units, with smaller units dissipating heat to the environment more rapidly due to increased surface area to volume ratio.

### 3.7. Inactivation Mechanisms

HPP is not known to directly damage nucleic acids. However high pressure is thought to either distort or destroy lipid membranes in bacteria by causing phase inversion. Leakage of cytosolic contents as a result of membrane integrity loss accounts for much of vegetative bacterial inactivation. A second mechanism of inactivation is via protein denaturation. On a molecular level, water forms a solvation cage around proteins. When high pressure is applied, more water is forced into this solvation cage resulting in changes in the tertiary and quaternary structure of proteins disrupting function. Non-enveloped viruses, such as norovirus and HAV, by definition do not have lipids associated with them. Therefore inactivation of food-borne viruses is via protein denaturation. 

### 3.8. Organoleptic Considerations

**Figure 1:**
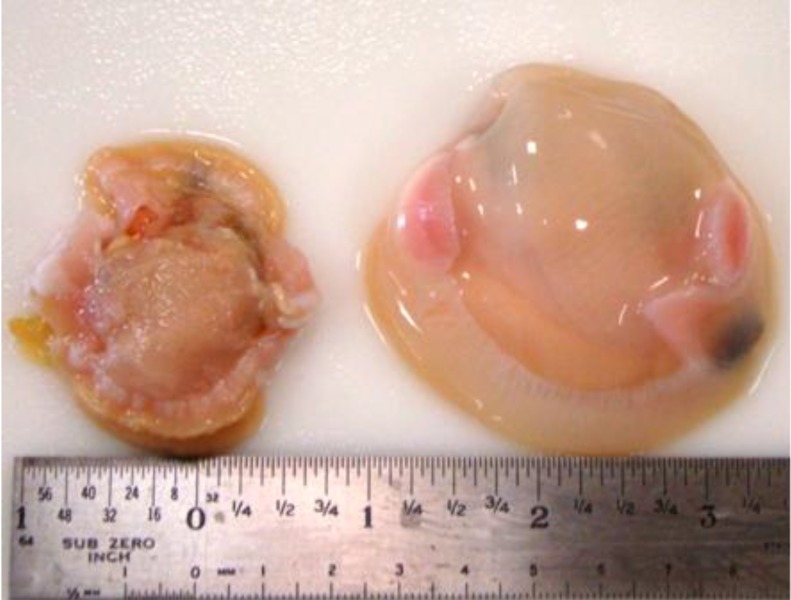
A hand-shucked clam (left; *Mercenaria mercenaria*) is compared to a high pressure processing (HPP)-treated clam (right). Note: this figure is reprinted with permission from [9]. Copyright Elsevier B.V. 2014.

The color, taste, texture, appearance and smell of raw oysters are of paramount concern to the industry and consumers alike. Currently there is a perception by industry that pressures above 300 MPa result in undesirable changes to oyster quality. It is true that the degree to which HPP changes a raw oyster’s characteristics is a function of the pressure applied and the temperature at which pressure is applied. Some whitening or blanching occurs when treating oysters at 600 MPa at room temperature but this is minimized when 600 MPa is performed at 5 °C. Overall appearance of oysters and clams on-the-half-shell is much better when shucked by HPP [96], since the bivalve meat is completely intact (see Figure 1). This differs from a hand-shucked oyster which can often be sliced by the shucking knife. There are reports that HPP can induce some firmness or chewiness in seafoods, but this change is relatively subtle and may be considered desirable, since firmness can be considered an attribute of freshness [13]. Juiciness and flavor are enhanced by HPP, since shellfish take up liquid from the surrounding liquor within the shell [44,97,98]. This attribute results in a “yield” increase since the shucked oyster becomes more voluminous due to absorption of liquor fluid. One drawback is that liquid taken up does not remain within the bivalve tissues over time. Thus an originally full jar of HPP-shucked oysters will be reduced in volume a week later. Also depending on what the oysters were feeding on when harvested, sometimes this liquid in the jar can be an unappealing yellow or greenish color.

Studies clearly indicate that HPP-treated oysters are well received after treatments at pressures that are higher than are currently used commercially to shuck oysters and inactivate vibrio [99]. For example, recent results (shown in Table 1) from an organoleptic study evaluating the taste of oysters has shown that oysters treated at 400–600 MPa at 6 °C and 300–500 MPa at 22 °C are actually preferred to manually-shucked oysters [100]. Another challenge for HPP-treated oysters is time between processing and consumption may be several days which may compromise their perceived quality as compared to a fresh-shucked oyster.

**Table 1:** Organoleptic analysis of HPP-treated ^#^ oysters * using untrained volunteers.

	Control	300 MPa 22 °C	400 MPa 22 °C	500 MPa 22 ° C	400 MPa 6 °C	500 MPa 6 °C	600 MPa 6 °C	*F* value (Sig *p* value)
**Appearance**	4.11 ± 1.6	5.46 ± 1.5	5.39 ± 1.4	5.20 ± 1.6	5.39 ± 1.5	5.45 ± 1.4	5.22 ± 1.7	5.78 (0.000)
**Texture**	4.54 ± 1.9	5.23 ± 1.7	5.36 ± 1.8	5.55 ± 1.6	5.20 ± 1.6	5.47 ± 1.5	5.43 ± 1.7	2.46 (0.024)
**Flavor**	4.64 ± 1.7	5.04 ± 1.8	5.05 ± 1.7	5.13 ± 1.7	4.86 ± 1.6	5.35 ± 1.6	5.27 ± 1.6	1.24 (0.287)
**Aroma**	4.90 ± 1.4	5.27 ± 1.3	5.04 ± 1.2	5.30 ± 1.3	5.27 ± 1.4	5.33 ± 1.3	5.33 ± 1.4	0.94 (0.469)
**Acceptability**	4.64 ± 1.6	5.14 ± 1.6	5.13 ± 1.6	5.28 ± 1.6	5.02 ± 1.5	5.53 ± 1.4	5.38 ± 1.6	2.05 (0.058)

^#^ All HPP treatments were for 5 min; * analysis performed using triploid oysters (*Crassostrea virginica*) obtained from Cape May NJ August 2013; ± represents standard deviation.

### 3.9. Challenges and Future Directions

There are a number of challenges to the widespread application of HPP to the shellfish industry. First HPP is relatively expensive with the minimal cost for a commercial scale unit being several hundred thousand US dollars. Thus to be economically viable, shellfish harvesters and processors must be relatively large in scale. Currently most shell aquaculture and fishing operations are too small to successfully amortize this expense. Development of a low cost pressure unit for limited shellfish quantities that is suitable for use near or at the point of consumption would be a boon for the shellfish industry and consumers alike. Another challenge is consumer resistance and regulations regarding in shell-shellfish. A closed shell is often used to judge that shellfish are alive and fresh. HPP kills shellfish, so consumers must be educated to the concept that these are safe and good to eat. In most jurisdictions, there are no longer prohibitions against sale of in-shell HPP-treated shellfish but regulations against theses may remain in some places.

Another potential challenge is that shellfish must be HPP-treated relatively quickly after harvest. If the liquor inside the shell dries out or is reduced in volume permitting air within the shell, as can happen after a day or two of cold storage, the shells may be cracked, or even crushed when pressure is applied due to air pockets under the shell (personal observation). Lastly, should HPP treatment become mainstream as a pathogen intervention technique for shellfish, it will be important that current hygienic quality standards for safe harvest remain in place. HPP should only be applied in addition to current hygienic standards, not in lieu of these standards or as a way to utilize shellfish grow under non-hygienic conditions.

## 4. Conclusions

High pressure processing is a viable nonthermal intervention for prominent food-borne pathogens associated with raw bivalve shellfish.
